# Homologous Over-Expression of Chain Length Determination Protein EpsC Increases the Molecular Weight of Exopolysaccharide in *Streptococcus thermophilus* 05-34

**DOI:** 10.3389/fmicb.2021.696222

**Published:** 2021-07-20

**Authors:** Zhengyuan Zhai, Shuxin Xie, Hongxing Zhang, Huaxi Yi, Yanling Hao

**Affiliations:** ^1^Beijing Advanced Innovation Center for Food Nutrition and Human Health, College of Food Science and Nutritional Engineering, China Agricultural University, Beijing, China; ^2^Key Laboratory of Functional Dairy, Co-constructed by Ministry of Education and Beijing Municipality, College of Food Science and Nutritional Engineering, China Agricultural University, Beijing, China; ^3^Department of Food Science, Beijing University of Agriculture, Beijing, China; ^4^College of Food Science and Engineering, Ocean University of China, Qingdao, China

**Keywords:** *Streptococcus thermophilus*, EpsC, homologous expression, molecular weight, yogurt, viscoelasticity

## Abstract

In *Streptococcus thermophilus*, EpsC is a polysaccharide co-polymerase which is involved in determining the chain length of EPS synthesized by the Wzx/Wzy-dependent pathway. Our previous study found that there was a positive correlation between transcription level of *epsC* and molecular weight of EPS in *S. thermophilus* 05-34. To further investigate the effects of EpsC on EPS biosynthesis, this gene was over-expressed in *S. thermophilus* 05-34 in this study. Reverse transcription qPCR and Western blotting confirmed the successful transcription and translation of *epsC* in 05-34, respectively. The yield of EPS was not affected by the over-expression of EpsC. Gas chromatography-mass spectrometry (GC-MS) showed that the monosaccharide composition was still composed of galactose and glucose in a molar ratio of 1.0:0.8, whereas high performance gel permeation chromatography (HPGPC) indicated that the molecular weight of EPS was increased from 4.62 × 10^5^ Da to 9.17 × 10^5^ Da by the over-expression of EpsC. In addition, *S. thermophilus* 05epsC which could produce higher molecular weight EPS improved the viscoelasticity and water-holding capacity of yogurt, but significantly reduced the level of syneresis in yogurt. In summary, these results indicated that homologous over-expression of EpsC in *S. thermophilus* could increase the molecular weight of EPS and improve the microrheological or physical properties of yogurt.

## Introduction

*Streptococcus thermophilus* is a homofermentative facultative anaerobe, which has been widely used as a starter culture for the manufacture of dairy products, e.g., yogurt and cheese (Pega et al., 2017; Vieira et al., 2019). In yogurt, the presence of exopolysaccharides (EPS) produced by *S. thermophilus* has been reported to improve viscosity and texture, increase resistance to mechanical handling and prevent syneresis (Broadbent et al., 2003; Cui et al., 2017). Therefore, the *in situ* production of EPS by *S. thermophilus* has the potential to decrease the amount of added ingredients and stabilizers, such as dairy proteins, starch, pectin, and hydrocolloids (Zeidan et al., 2017). The replacement of these additives would lead to a clean label and a reduced production cost. Moreover, some EPSs produced by *S. thermophilus* have antibacterial, antioxidant, anti-inflammatory, and immunomodulating activities (Marcial et al., 2013; Li and Shah, 2014; Zhang et al., 2016). Consequently, EPS production is one of the most important and attractive properties of *S. thermophilus* strains.

Based on monosaccharide composition, the bacterial EPS are classified as homopolysaccharides (HoPS) and heteropolysaccharides (HePS). HoPS consist of only one type of monosaccharide. HePS are complex polymers with a backbone of repeating subunits that consists of three to eight monosaccharides, such as glucose, galactose, and rhamnose (Zeidan et al., 2017). HePS often differ by monosaccharide ratios, polymerization degree, charge, and linkages between units (Torino et al., 2015). The diverse EPS structures lead to the different functions of EPS, which are further connected to the rheological properties of dairy products. For example, high-molecular mass EPS produced by *S. thermophilus* can improve the viscosity of acidified milk (Faber et al., 1998; Mende et al., 2012). A stiff and linear EPS produced by *S. thermophilus* 2104 led to higher viscosity than other branched and flexible EPS (Gentès et al., 2011). In addition, anionic EPS led to higher viscosity and shear stability in stirred acid milk products (Mende et al., 2016). Therefore, a better understanding of the EPS structure–function relationship might provide new strategy for developing EPS with desirable characteristics and improving the rheological properties of dairy products.

The molecular structure of EPS has been proved to be regulated by a specific gene cluster (Zeidan et al., 2017). Generally, the *eps* cluster of *S. thermophilus* can be divided in three parts: the first region includes the regulatory genes of EPS production (*epsA, epsB*) and chain length determination genes (*epsC, epsD*); the second region is associated with biosynthesis of repeating units of EPS (*epsE, epsI*, and *epsJ*); the third region is responsible for polymerization and export of repeating units (*epsH, epsK*) (Zeidan et al., 2017; Wang et al., 2020). Notably, the chain length determination gene *epsC* and *epsD* are involved in polysaccharide assembly (Cui et al., 2017). When intracellular C-terminal portion of CpsC (an EpsC homolog) is deleted, long are produced by *Streptococcus agalactiae* (Toniolo et al., 2015). In *Xanthomonas campestris*, higher molecular weight xanthan is produced when the polysaccharide co-polymerase GumC (an EpsC homolog) is over-expressed (Galván et al., 2013). In addition, GumC over-expression increased xanthan viscosity. In our previous study, the transcription level of *epsC* showed a 2.7-fold up-regulation in *S. thermophilus* 05-34 under the optimal fermentation condition (Li et al., 2016). Meanwhile, the molecular weight of EPS was increased by nine times compared with that in the non-optimal fermentation condition. However, the relationship between EpsC expression level and molecular structure of EPS remains elusive in *S. thermophilus*. In this study, homologous over-expression was employed to investigate if the expression level of EpsC could affect the molecular weight of EPS and the microrheology of yogurt.

## Materials and Methods

### Bacterial Strains, Plasmids, and Growth Conditions

The bacterial strains and plasmids used in this study are listed in Table 1. *Streptococcus thermophilus* and *Lactobacillus delbrueckii* subsp. *bulgaricus* were grown under static conditions at 37°C in de Man-Rogosa-Sharpe (MRS) broth for 24 h. As a host for gene cloning, *Lactococcus lactis* was routinely grown at 30°C in M17 medium (Oxoid, Unipath, Basingstoke, United Kingdom) containing 0.5% wt/vol glucose (GM17) for 24 h. When necessary, medium was supplemented with 5 μg/mL chloramphenicol for *S. thermophilus* and *Lc. lactis*.

**TABLE 1:** Bacterial strains and plasmids used in this study.

**Strain or plasmid**	**Relevant phenotype or genotype^*a*^**	**Source**
**Bacterial strains**		
*S. thermophilus* 05-34	EPS-producing strain, isolated from Tibetan kefir grains	Laboratory stock
*Lc. lactis* NZ9000	*Lc. lactis* MG1363 *pepN::nisRK*	De Ruyter et al., 1996
*Lb. delbrueckii* subsp. *bulgaricus* 05-22	Non-EPS-producing strain isolated from commercial starter culture	Laboratory stock
*Lc. lactis* epsC	*Lc. lactis* NZ9000 harboring pSlpA-epsC	This work
*Lc. lactis* epsCh_6_	*Lc. lactis* NZ9000 harboring pSlpA-epsCh_6_	This work
*S. thermophilus* 05CK	*S. thermophilus* 05-34 harboring pSlpA-8148	Wang et al., 2020
*S. thermophilus* 05epsC	*S. thermophilus* 05-34 harboring pSlpA-epsC	This work
*S. thermophilus* 05epsCh_6_	*S. thermophilus* 05-34 harboring pSlpA-epsCh_6_	This work
**Plasmids**		
pSlpA-8148	Cm^*r*^, constitutive expression vector carrying the *PslpA* promoter	Wang et al., 2020
pSlpA-epsC	pSlpA-8148 derivative containing *bmrR* gene	This work
pSlpA-epsCh_6_	pSlpA-8148 derivative containing *epsC* gene with a 3′-sequence encoding a C-terminal His-tag	This work

*^*a*^Cm^*r*^, chloramphenicol resistance.*

### DNA Manipulation Techniques

Genomic DNA from *S. thermophilus* 05-34 was prepared by lysozyme pretreatment and the genomic DNA Extraction Kit (Tiangen, Beijing, China). Mini-prep plasmid isolations from *Lc. lactis* were performed using the E.Z.N.A^TM^ Plasmid Mini Kit I (OMEGA Bio-tek Inc., Doraville, GA, United States). Standard PCR was carried out using Q5^TM^ High-Fidelity DNA polymerase following the manufacturer’s instructions (NEB, Beijing, China). DNA digestion with restriction endonucleases and DNA ligation were performed according to the manufacturer’s instructions (NEB). The electroporation of *Lc. lactis* NZ9000 was preformed according to previously described procedures (Holo and Nes, 1989). Primers used in this study were designed using PRIMER V5 software (PREMIER Biosoft International, Palo Alto, CA) and synthesized by Sangon Biotech (Beijing, China). DNA sequencing was performed by Sangon Biotech and the results were further analyzed with the DNAMAN software package (Lynnon Biosoftware, Vaudreuil, QC, Canada).

### Construction of the Recombinant Strain *S. thermophilus* 05epsC

The *epsC* was amplified from genomic DNA of *S. thermophilus* 05-34 using the primer pair: F*-epsC*: 5′-CATGCCATGGGGAATCAAGATAACAC-3′ and R-*epsC* 5′- CGCGAGCTCTTAAATTTTATCTGTATC-3′. Restriction sites *Nco*I and *Sac*I used for subsequent cloning are underlined. The PCR product digested by *Nco*I and *Sac*I was inserted into the corresponding sites of pSlpA-8148 at the downstream of *P*_*slpA*_ promoter. Subsequently, the ligation mixture was transformed into *Lc. lactis* NZ9000 by electroporation. *Lc. lactis* transformants were selected on GM17 plates containing 5 μg/mL chloramphenicol. Plasmids were isolated from *Lc. lactis* transformants and further verified by DNA sequencing. Subsequently, the resulting plasmid pSlpA-epsC was transformed into *S. thermophilus* 05-34 by electroporation in a 0.2 cm cuvette at 1.5 kV, 25 mF, and 200 Ω (Marciset and Mollet, 1994). The recombinant strain was designated as *S. thermophilus* 05epsC. Moreover, plasmid pSlpA-epsC in *S. thermophilus* 05epsC was extracted and verified by DNA sequencing.

### Transcriptional Analysis of *epsC* in *S. thermophilus* 05epsC

*Streptococcus thermophilus* 05epsC and the control strain *S. thermophilus* 05CK were grown in 10 mL MRS broth (initial pH 7.0) at 37°C for 30 h. Cells were harvested by centrifugation at 6,000 × *g* for 10 min. The total RNA was isolated using RNAprep pure cell/bacteria kit following the manufacture’s instruction (Tiangen, Beijing, China). Subsequently, 1 μg of total RNA was used as the template for reverse transcription. And cDNA was obtained using PrimeScript II 1st strand cDNA synthesis kit (Takara). Real-time quantitative PCR (qPCR) was performed using SuperReal PreMix Plus SYBR Green kit (Tiangen, Beijing, China) on LightCycler 480 real-time thermocycler (Roche Diagnostics, Meylan, France). The primers used for qPCR are listed in Supplementary Table 1. Primer specificity was assessed by examination of the melting curve at the end of amplification. Gene expressions were calculated by the 2^–ΔΔ*Ct*^ method by using the 16S rRNA as the reference gene (Schmittgen and Livak, 2008).

### Western Blotting Assay

To confirm the over-expression of *epsC* in *S. thermophilus* 05-34 by Western blotting, the *epsC* gene was amplified by PCR using primers F-*epsC* and R-*epsC*h_6_ (Supplementary Table 1), thereby introducing a 6× His tag coding sequence to the 3′-end. This amplicon was cloned into pSlpA-8148, and then transformed into *S. thermophiles* 05-34, resulting in the recombinant strain *S. thermophilus* 05epsCh_6_. *S. thermophilus* 05CK and 05epsCh_6_ were grown under static conditions at 37°C in MRS broth supplemented with 5 μg/mL chloramphenicol for 30 h prior to protein extraction. Protein extraction and Western blot were performed as previously described (Wang et al., 2020).

### Isolation and Purification of EPS

For EPS isolation, overnight cultures of *S. thermophilus* 05epsC and 05CK were inoculated (2% v/v) into 1 L sterilized reconstituted skim milk medium (RSM, 10% w/v) and grown for 30 h at 37°C. The EPS extraction were performed as previously described (Qin et al., 2011). Briefly, cultures were heated at 100°C for 15 min and centrifuged at 6,000 g for 15 min to remove precipitates. And then proteins in the supernatant were precipitated by adding trichloroacetic acid (TCA) at final concentration of 4% (v/v). After 2 h incubation at 4°C, the supernatant was collected by centrifugation at 6,000 g for 15 min. To precipitate crude EPS, three volumes of cold absolute ethanol was mixed with one volume of supernatant. After 24 h incubation at 4°C, the pellet containing EPS was obtained by centrifugation at 6,000 g for 20 min. Crude EPS was dialyzed with a membrane of molecular weight cut-off (MWCO) 8–10 kDa and then lyophilized. The EPS was purified by a Sepharose CL-6B gel chromatography column (1.6 × 80 cm, Pharmacia, Piscataway, NJ, United States) as previously described (Li et al., 2016). Each 10 mL of elution was collected automatically. Elution curve was plotted by phenol-sulfuric acid assay (detected at 490 nm). The yield of EPS was quantified using the phenol-sulfuric method using glucose as a standard (Dubois et al., 1956). Concentration of protein and nucleic acid of purified fraction was determined spectroscopically at 280 and 260 nm, respectively.

### Molecular Weight and Monosaccharide Composition of EPS

The average molecular weight (Mw) of EPS was measured by high performance gel permeation chromatography (HPGPC). The HPGPC system consisted of Agilent 1260 series HPLC system (Agilent, United States) and Shodex SUGAR KS series containing KS-804 and KS-805 columns (Shodex Co., Tokyo, Japan). The purified EPS was detected using refractive index detector (RI-2000, Waters, United States) according to the method which we used previously (Wang et al., 2020). Briefly, 40 μg purified EPS was eluted with 20 mM NaCl at a flow rate of 0.8 mL/min. Dextrans of known Mw (5,000, 11,600, 23,800, 48,600, 80,900, 148,000, 273,000, 409,800, and 667,800 Da, Sigma–Aldrich, St. Louis, MO, United States) were used to calibrate the Mw of sample. Data processing was performed with Cirrus^TM^ GPC software version 3.0 (Agilent Technologies, Santa Clara, CA, United States).

The monosaccharide composition was determined by gas chromatography coupled with mass spectrograph (GC-MS) as previously described (Li et al., 2016). Briefly, 2 mg purified EPS was hydrolyzed with 2 mL trifluoroacetic acid (TFA) at 120°C for 90 min. The hydrolysate was neutralized to pH 7.0 with 2 M NaOH and evaporated. The hydrolysate was reduced with 30 mg/mL NaBH_4_ for 8 h at room temperature. The excess NaBH_4_ was decomposed with acetic acid and removed by repeated evaporation to dryness with the addition 0.1% (v/v) methanol. Afterward, the dry hydrolysate was dissolved in 1 mL acetic anhydride, and then the mixture was incubated at 100°C for 60 min. After the reaction, 3 mL of methylbenzene was added and the product was dried with a rotary evaporator. A mixture of chloroform (1 mL) and deionized water (1 mL) was added to the sample, followed by vortexing. The organic phase was subsequently analyzed on a GC 7890A platform (Agilent Technologies, United States), equipped with an HP-5 column (30 m × 0.25 mm × 0.25 μm) and a mass spectrum (MS) analyzer. The conditions for GC-MS were used as follows: initial column temperature was set at 120°C with a rate of 3°C/min to reach 250°C and then held at 250°C for 5 min. Samples were injected into the column with N_2_ as the carrier gas at a flow rate of 1 mL/min. The following monosaccharides were derivatized as standards: rhamnose, fucose, arabinose, xylose, mannose, glucose, galactose. The monosaccharide composition of EPS was determined by comparison with the retention time of monosaccharide standards.

### Microrheological Properties of Yogurt Produced by *S. thermophilus* 05epsC

The passive microrheology of yogurt was measured by diffusing-wave spectroscopy (DWS, LAB 6 Rheolaser MASTER, Formulaction SAS, I’Union, France). The production of yogurt was carried out as described previously (Qin et al., 2011). Ultra-high temperature treated (UHT) whole bovine milk was purchased from San Yuan Foods Company (Beijing, China) through a local distributor. This milk contained 3.1% fat, 2.9% protein, and 8.1% (w/w) non-fat milk solid. Non-EPS-producing *Lb. delbrueckii* subsp. *bulgaricus* 05-22 was used with *S. thermophilus* strains as starter cultures in this study. The experimental group yogurt was inoculated (1.5% v/v) with *S. thermophilus* 05epsC and *Lb. delbrueckii* subsp. *bulgaricus* 05-22 in a ratio of 1:1, while the control group was inoculated (1.5% v/v) with *S. thermophilus* 05CK and *Lb. delbrueckii* subsp. *bulgaricus* 05-22. Immediately after inoculation, 20 mL aliquots of the samples were transferred into optical glass cuvettes of LAB 6 Rheolaser MASTER. Gel formation was observed at 42°C. The observation and data collection were continued for over 6 h. The scatterer mobility, in terms of speed and displacement, enabled the mean square displacement (MSD) of the particles vs. decorrelation time to be plotted, which was directly related to the viscoelastic properties. Elasticity index (EI) and macroscopic viscosity index (MVI) were also calculated as described previously (Sun et al., 2018). The data, including MSD curve, EI and MVI, were collected and calculated using the software 1.4.0.0 incorporated in the instrument.

### Physical Properties of Yogurt Produced by *S. thermophilus* 05epsC

The production of yogurt was carried out as described above. All batches were incubated at 42°C until pH 4.6 was reached. Acidity was measured by a titrimetric method with 0.1 mol/L NaOH as described previously (Qin et al., 2011). For water-holding capacity (WHC), 20 g sample were centrifuged at 500 g for 10 min at 4°C. The supernatant was then collected by pipette and weighed. The water holding capacity was determined in triplicate and calculated as follows: $\text{WHC}=\left(1-\frac{M_{S}}{M_{Y}}\right)\times 100\%$. WHC is the water holding capacity of the sample, M_*S*_ is the supernatant after centrifugation and M_*Y*_ is the mass of the weighed yogurt. Whey separation was measured by weighing 100 g of yogurt into a funnel with one layer of 200 mesh nylon fabric. Whey was collected and weighed (% w/w) after 3 h at 6°C as described previously (Qin et al., 2011).

### Statistical Analysis

Data were analyzed using GraphPad Prism 6 software for Windows (GraphPad Software, Inc., La Jolla, CA, United States). When two groups were compared, an unpaired Student *t*-test with Welch’s correction was used to calculate *P*-values.

## Results

### Homologous Over-Expression of EpsC

**FIGURE 1:**
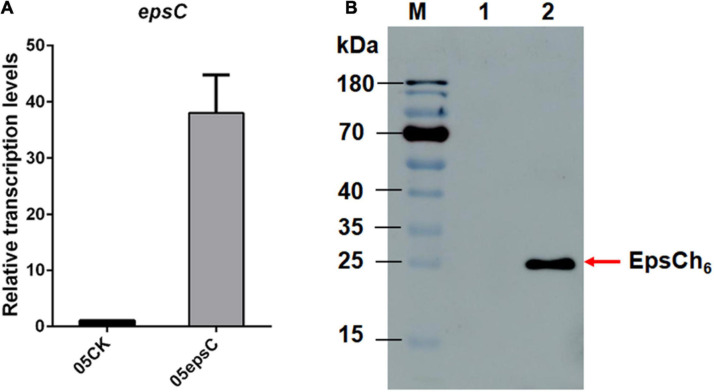
The over-expression of *epsC* in *S. thermophilus* 05-34. **(A)** RT-qPCR analysis of the transcription level of *epsC* in *S. thermophilus* 05epsC. The fold change calculated was relative to the transcript levels in *S. thermophilus* 05epsC compared with that in 05CK. They were normalized using 16S rRNA gene as an internal control. Data are reported as mean ± SD from three independent experiments. **(B)** Detection of the over-expression of EpsC in *S. thermophilus* by Western blotting. M: PageRuler Prestained Protein Ladder (Thermo Fisher Scientific, Waltham, MA, United States). Lane 1: Protein sample extracted from *S. thermophilus* 05CK; Lane 2: Protein sample extracted from *S. thermophilus* 05epsCh_6_.

For homologous over-expression, the expected 693 bp *epsC* gene was amplified, purified and cloned into constitutive expression vector pSlpA-8148 to generate recombinant plasmid, designated as pSlpA-epsC. DNA sequencing showed that gene *epsC* in pSlpA-epsC was 100% identity to the *epsC* gene from *S. thermophilus* 05-34 (DIS_02530; GenBank Accession No. QFLC00000000). RT-qPCR revealed that the transcription level of *epsC* in *S. thermophilus* 05epsC was 38.04 ± 6.74 fold higher than that in the control strain *S. thermophilus* 05CK (Figure 1A). Western blotting assay with anti His-Tag mouse monoclonal antibody further confirmed the production of a 26-kDa protein in *S. thermophilus* 05epsCh_6_, while no corresponding band was observed in *S. thermophilus* 05CK (Figure 1B). These results indicated that gene *epsC* was successfully over-expressed in *S. thermophilus* 05-34.

### Isolation and Purification of EPS Produced by *S. thermophilus* 05epsC

*Streptococcus thermophilus* 05epsC and 05CK were cultivated in 10% reconstituted skim milk (RSM) at 37°C for 30 h, respectively. Then EPS from RSM samples was isolated, purified and quantified. According to the elution profile of EPS from *S. thermophilus* 05epsC, there was only one single and relatively symmetrical peak at OD_490 *nm*_ in fraction No. 7 (Supplementary Figure 2), while the peak of the elution profile in the control group appeared in fraction No. 9. These results suggested that over-expression of *epsC* in *S. thermophilus* 05-34 increased the average molecular weight of EPS. The ultraviolet spectrum of purified EPS fraction showed no absorption at 260 or 280 nm, indicating that there was no nucleic acids or protein contamination in EPS samples. Based on the glucose standard (Supplementary Figure 1), the yield of EPS produced by *S. thermophilus* 05epsC and 05CK was 85 ± 3 mg/L and 73 ± 9 mg/L, respectively. There is no significant difference in the yield of EPS between 05epsC and 05CK (*P* = 0.0936, Table 2).

**TABLE 2:** The characteristics of EPS produced by *S. thermophilus* 05CK and 05epsC.

	**Strain**	
	***S. thermophilus* 05CK**	***S. thermophilus* 05epsC**
Yield (mg/L)	73 ± 9	85 ± 3
Monosaccharide composition	Galactose and glucose	Galactose and glucose
Molar ratio (Gal/Glc)	1.25	1.25
Molecular weight (Da)	4.62 × 10^5^	9.17 × 10^5^

### Monosaccharide Composition and Molecular Weight of EPS Produced by *S. thermophilus* 05epsC

**FIGURE 2:**
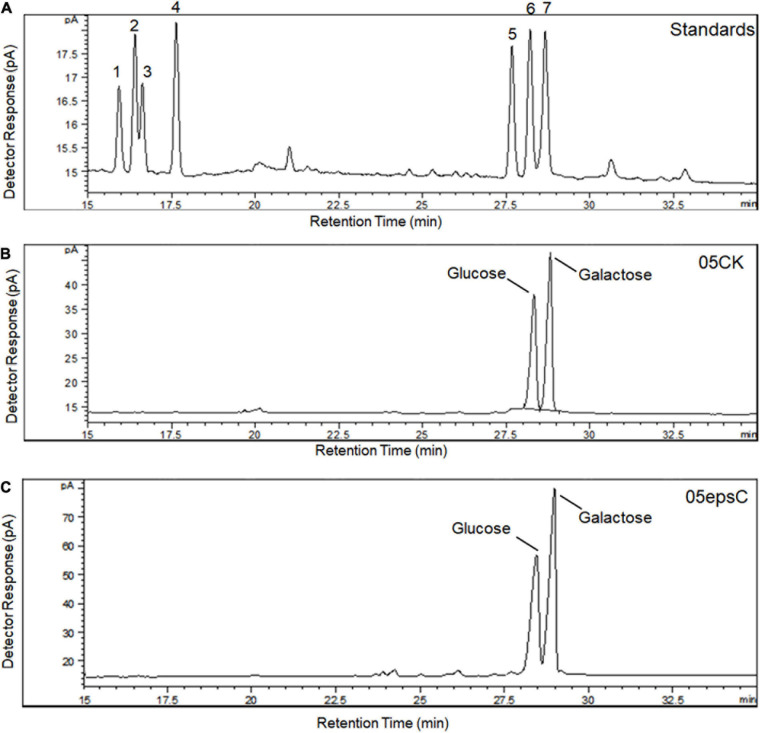
Monosaccharide composition of hydrolyzed EPS from *S. thermophilus* 05epsC and 05CK by GC-MS analysis. **(A)** Monosaccharide standards: (1) rhamnose; (2) fucose; (3) arabinose; (4) xylose; (5) mannose; (6) glucose; (7) galactose. **(B)** Hydrolyzed EPS from *S. thermophilus* 05CK. **(C)** Hydrolyzed EPS from *S. thermophilus* 05epsC.

**FIGURE 3:**
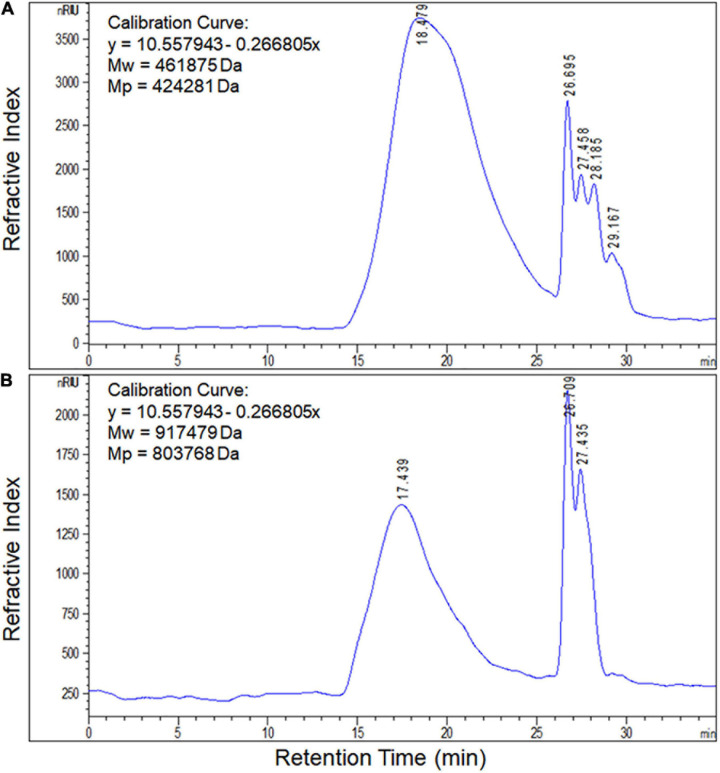
Molecular weight of EPS produced by *S. thermophilus* 05epsC **(A)** and 05CK **(B)**. According to the linear regression equation drawn through dextran standards, the molecular weight of EPS samples was determined based on their retention time (X-axis) using gel permeation chromatography (GPC). Calibration curve was y = 10.557943 – 0.266805x. Mw, Average molecular weight; Mp, Peak molecular weight.

Based on the retention time of different monosaccharide standards, the monomer analysis by GC-MS indicated that the EPS produced by *S. thermophilus* 05epsC and 05CK were both composed of galactose and glucose in an approximate ratio of 1.0:0.8 (Figure 2). The average molecular weight of EPS produced by 05CK was 4.62 × 10^5^ Da, which was determined by HPGPC. However, the average molecular weight of EPS produced by *S. thermophilus* 05epsC was increased to be 9.17 × 10^5^ Da, which was 1.98-fold higher than that of 05CK (Figure 3). In this study, the yield and monosaccharide composition of EPS in 05-34 was not affected by over-expression of *epsC*.

### The Microrheology of Yogurt Fermented by *S. thermophilus* 05epsC

**FIGURE 4:**
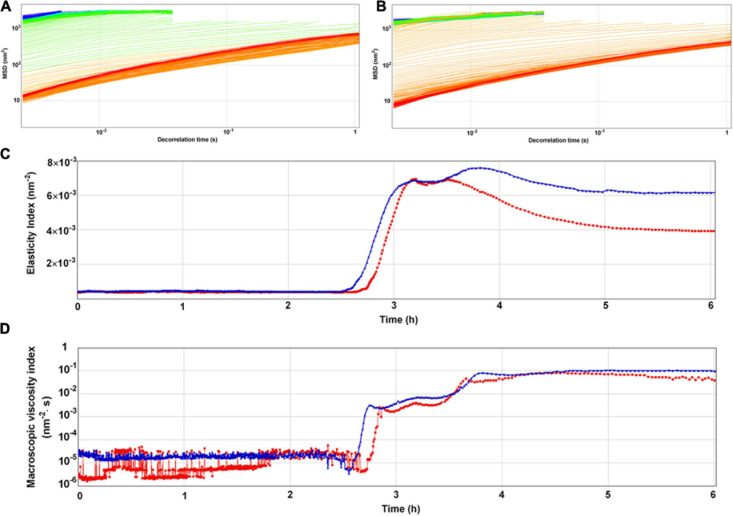
The representative mean square displacement (MSD) curves, elasticity index (EI), and macroscopic viscosity index (MVI) of yogurt produced by *S. thermophilus* 05epsC. **(A)** MSD of yogurt produced by *S. thermophilus* 05CK; **(B)** MSD of yogurt produced by 05epsC; **(C)** EI of yogurt produced by 05epsC and 05CK; **(D)** MVI of yogurt produced by 05epsC and 05CK; Blue triangle: yogurt produced by 05epsC; Red square: yogurt produced by 05CK.

The entire fermentation of yogurt was monitored using DWS which has been widely applied to milk protein and polysaccharide mixed systems (Alexander et al., 2008). The time-dependent MSD profiles for all samples showed linear motion, indicating typical free diffusion of particles in the yogurts during fermentation (Figures 4A,B). After 4 h incubation, particles traveled less displacement distance in the yogurt made with *S. thermophilus* 05epsC than in the control during the same decorrelation time. For instance, when decorrelation time was 0.1 s after 6 h incubation, MSD of particles in the yogurt made with *S. thermophilus* 05epsC was 141 ± 1.41 nm^2^. Meanwhile, MSD of particles in the control was 235 ± 1.41 nm^2^ (Table 3). Particles traveled less displacement distance in the yogurt made with *S. thermophilus* 05epsC indicated that they have a lower particle speed and higher macroscopic viscosity (Tisserand et al., 2016). Therefore, the MVI of yogurt produced by *S. thermophilus* 05epsC was significantly higher than that of the control after 5 h incubation (Table 3). In addition, the elasticity index of yogurt produced by *S. thermophilus* 05epsC was significantly higher than that of the control after 4 h incubation (Figure 4C and Table 3). These results indicated that EPS with higher molecular weight produced by *S. thermophilus* 05epsC could increase the viscoelasticity of yogurt.

**TABLE 3:** The MSD, macroscopic viscosity index, and elasticity index of yogurt produced by *S. thermophilus* 05epsC or 05CK at 4, 5, and 6 h.

	**Incubation time (h)**	**Samples**	
		***S. thermophilus* 05CK+*Lb*^*c*^**	***S. thermophilus* 05epsC+*Lb***
MSD^*a*^ (nm^2^)	4	153 ± 7.07	120.5 ± 6.36*^d^
	5	218. ± 1.41	137.5 ± 2.12**
	6	235 ± 1.41	141 ± 1.41**
MVI^*b*^ (10^–2^ nm^–2^⋅s)	4	4.01 ± 0.01	5.71 ± 1.00
	5	6.43 ± 0.41	10.34 ± 0.65*
	6	4.45 ± 0.88	9.63 ± 0.66*
EI (10^–3^ nm^–2^)	4	5.85 ± 0.14	7.23 ± 0.23*
	5	4.1 ± 0.08	6.39 ± 0.21**
	6	3.86 ± 0.11	6.27 ± 0.16**

*^*a*^MSD, mean square displacement, the decorrelation time was 0.1 second.*

*^*b*^MVI, macroscopic viscosity index.*

*^*c*^“*Lb*” indicated *Lb. delbrueckii* subsp. *bulgaricus* 05-22.*

^*d*^An unpaired Student *t*-test with Welch’s correction was used to calculate *P*-values.

*P < 0.05, **P < 0.01, compared to yogurt produced by 05CK at each time point, *n* = 2.

### The Physical Properties of Yogurt Fermented by *S. thermophilus* 05epsC

The physical properties of yogurt after fermentation were also measured and listed in Table 4. There was no significant difference in titratable acidity between the experimental group and the control group (*P >* 0.05), which suggested that EPS produced by *S. thermophilus* 05epsC had no adverse effect on final acidity. However, the WHC of yogurt made with *S. thermophilus* 05epsC was slightly higher than that of the control group (*P* < 0.05). It is worth to note that the whey separation of yogurt made with *S. thermophilus* 05epsC was 36.70 ± 2.30% (w/w), which was significantly lower than that of the control group (40.80 ± 0.93% w/w). These results suggested that higher molecular weight EPS produced by *S. thermophilus* 05epsC could significantly reduce the level of syneresis in yogurt.

**TABLE 4:** The physical properties of yogurt produced by *S. thermophilus* 05epsC or 05CK.

	**Yogurt**	
	***S. thermophilus* 05CK+*Lb*^*a*^**	***S. thermophilus* 05epsC+*Lb***
pH (10°C)	4.41 ± 0.03	4.41 ± 0.01
Titratable acidity (°T)	82.51 ± 0.52	82.68 ± 0.59
WHC (% w/w)	77.02 ± 0.80	81.98 ± 1.71*^b^
Whey separation (% w/w)	40.80 ± 0.93	36.70 ± 2.30*

*^*a*^“*Lb*” indicated *Lb. delbrueckii* subsp. *bulgaricus* 05-22.*

^*b*^An unpaired Student *t*-test with Welch’s correction was used to calculate *P*-values.

*P < 0.05, compared to yogurt produced by 05CK, *n* = 3.

## Discussion

In *S. thermophilus*, EpsC is a PCP which is involved in determining the chain length of EPS synthesized by the Wzx/Wzy-dependent pathway (Cui et al., 2017). Topological analysis with TMHMM program revealed that EpsC from *S. thermophilus* 05-34 is a cytoplasmic membrane protein and contains two transmembrane helices located in amino acid units 25–44 and 181–198 (Supplementary Figure 3A). Meanwhile, the region between amino acid units 48 and 180 was located out of the cell membrane, suggesting that nearly 60% of the protein is located on the outer membrane. Sequence alignment revealed that EpsC from *S. thermophilus* 05-34 showed 92.61% similarity at the amino acid level with tyrosine-protein kinase transmembrane modulator EpsC from *S. thermophilus* Sfi6. Secondary structure prediction of EpsC showed that it contains four beta strands and four alpha helices (Supplementary Figure 3B). The arrangement of secondary structure elements indicated that EpsC has a fold similar to the base domain of PCP family proteins, which could be involved in chain-length determination during polysaccharide biosynthesis (Morona et al., 2009). Our previous study indicated that there was a positive correlation between transcription level of *epsC* and molecular weight of EPS in *S. thermophilus* 05-34 (Li et al., 2016). In this study, we found that the average molecular weight of EPS increased from 4.62 × 10^5^ Da to 9.17 × 10^5^ Da after homologous over-expression of *epsC* in *S. thermophilus* 05-34. However, the yield and monosaccharide composition of EPS were not affected by over-expression of *epsC*. These results confirmed the role of EpsC in chain length determination during EPS biosynthesis.

Previous studies provided some evidence that viscosity of yogurt was relevant to the molecular weight of EPS produced by the *S. thermophilus* strains (Faber et al., 1998; Xu et al., 2021). For instance, the average molecular weight of EPS produced by *S. thermophilus* Sts and Rs was 3.7 × 10^3^ kDa and 2.6 × 10^3^ kDa, respectively. The viscosity of fermented milk produced by *S. thermophilus* Sts was 126 ± 15 s, which was 3.2-fold higher than that of fermented milk produced by Rs. These results gave a conclusion that EPS with higher molecular weight might contribute to the higher viscosity of products (Faber et al., 1998). Although EPS from these two strains have the same branched heptasaccharide repeating unit, the differences between *S. thermophilus* Sts and Rs in growth, fermentation rate or post-acidification might also affect the viscosity of stirred milk cultures. In this paper, *S. thermophilus* 05epsC and 05CK was derived from the same strain 05-34. The MVI of yogurt produced by *S. thermophilus* 05epsC was significantly higher than that of the control after 5 h incubation. Notably, the viscosity of yogurt can be represented by MVI (Rohart et al., 2016; Bai et al., 2020). The viscosity of fermented milk could be increased by the production of high molecular weight EPS (Hamet et al., 2015). In RSM, over-expression of EpsC increased the molecular weight of EPS, but did not affect the monosaccharide composition and yield. Both RSM and yogurt are milk-based medium and similar in nutritional composition. To further confirm the EpsC over-expression in yogurt, the transcription level of *epsC* in yogurt has been determined by RT-qPCR. The transcription level of *epsC* in *S. thermophilus* 05epsC in yogurt was 42.22 ± 7.13 fold higher than that in the control strain 05CK (Supplementary Figure 4). Therefore, the differences between the yogurt fermented by 05epsC and 05CK was mainly caused by the EPS with different molecular weight.

Generally, EPS can modify yogurt microstructure by preventing casein particles to form large aggregates (Han et al., 2016). Casein particles interconnect to form filaments with the bigger interspace, resulting in lower whey separation. In skim milk gels, the levels of syneresis were decreased by increasing the molecular weight of beta-glucan from 70 to 250 × 10^3^ Da (Lazaridou et al., 2008). This is in consistent with our finding that higher molecular weight EPS produced by *S. thermophilus* 05epsC could significantly reduce the level of syneresis in yogurt. Therefore, we speculated that higher molecular weight EPS produced by *S. thermophilus* 05epsC may enhance the structural integrity of the protein-polysaccharide network and the entrapment of the whey in yogurt. To our knowledge, this is the first report about that over-expression of EpsC increased the molecular weight of EPS in *S. thermophilus* and improved the viscoelasticity and stability of yogurt. These findings will provide new insight into the structure-function relationship of EPS produced by *S. thermophilus*. And modulation of *epsC* expression could be a strategy for developing EPS with desirable molecular weight and improving the microrheology of dairy products.

## Data Availability Statement

The original contributions presented in the study are included in the article/Supplementary Material, further inquiries can be directed to the corresponding author/s.

## Author Contributions

YH and ZZ designed the study, wrote the manuscript, and revised the manuscript. SX and ZZ performed the experiments. HZ and HY analyzed and evaluated the data. All authors read and approved the final manuscript.

## Conflict of Interest

The authors declare that the research was conducted in the absence of any commercial or financial relationships that could be construed as a potential conflict of interest.
