# Supplementary material for: Homologous Over-Expression of Chain Length Determination Protein EpsC Increases the Molecular Weight of Exopolysaccharide in Streptococcus thermophilus 05-34
Source: Front Microbiol. 2021 Jul 20;12:696222. doi: 10.3389/fmicb.2021.696222 (PMC8329376; doi:10.3389/fmicb.2021.696222)
Supplement: Supplementary file 1 [file Data_Sheet_1.docx]

Supplementary Material

**Table S1.** Primers used for vector construction and RT-qPCR

| Primer name | Sequence of primers used ^a^ |
| --- | --- |
| F*-epsC* | 5' CATGCCATGGGGAATCAAGATAACAC 3' |
| R*-epsC* | 5' CGCGAGCTCTTAAATTTTATCTGTATC 3' |
| R-*epsC*h_6_ | 5' CGAGCTCTTAATGATGATGATGATGATGAATTTTATCTG  TATCAGG 3' |
| F-*epsC*-RT | 5' ATGTCACAACGCTCGAAG 3' |
| R-*epsC*-RT | 5' GGCGAACACGATCATCT 3' |
| F-16s | 5' GATGGACCTGCGTTGTAT 3' |
| R-16s | 5' TTCCCTACTGCTGCCTC 3' |

^a^ The *Nco*I and *Sac*I restriction sites were underlined, respectively.


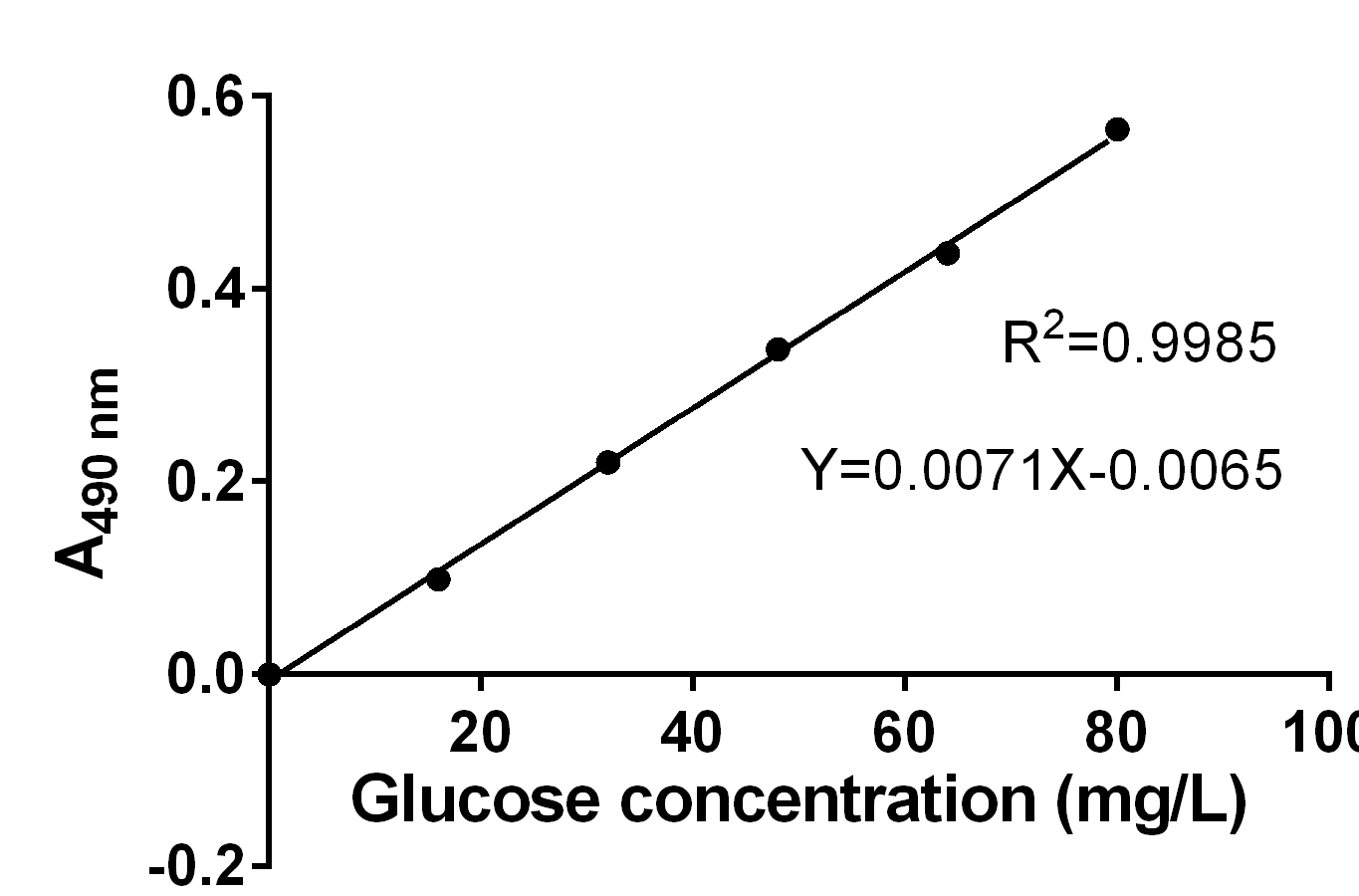


**Figure S1**. Glucose standard curve for EPS quantification.


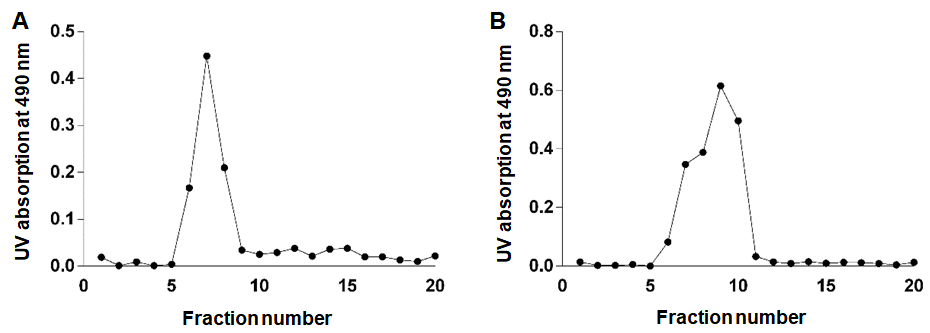


**Figure S2** The elution curves of EPS produced by *S. thermophilus* 05epsC (A) and *S. thermophilus* 05CK (B). Each 10 mL of elution was collected automatically. Elution curve was plotted by phenol-sulfuric acid assay (detected at 490 nm).


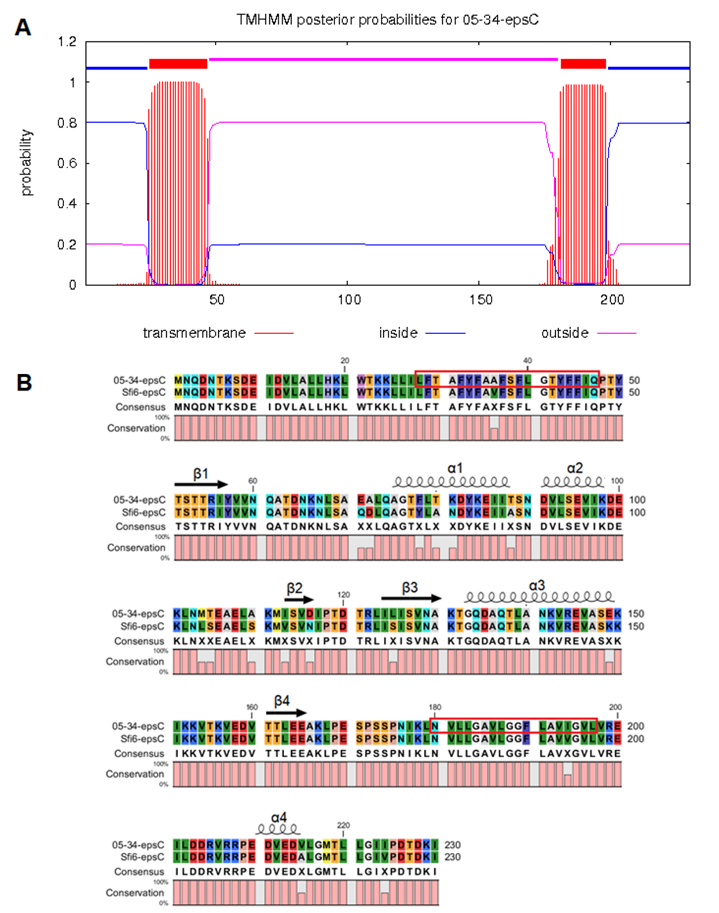


**Figure S3** Structure prediction and sequence alignment of EpsC from *S. thermophilus* 05-34 with homologous chain length determination protein from *S. thermophilus* Sfi6. (A) Prediction of transmembrane regions and orientation of EpsC by the program TMHMM. The x-axis represents the location of amino acids, and the y-axis represents the probability of membrane-spanning. When the probability value is above 0.5, the corresponding location of the amino acid is the predicted transmembrane region. Sequence alignment was performed with EpsC homologs from *S. thermophilus* Sfi6 and 05-34 using ClustalX 2.1(Larkin et al., 2007) with default settings and visualized in CLC Sequence Viewer 7.8.1.


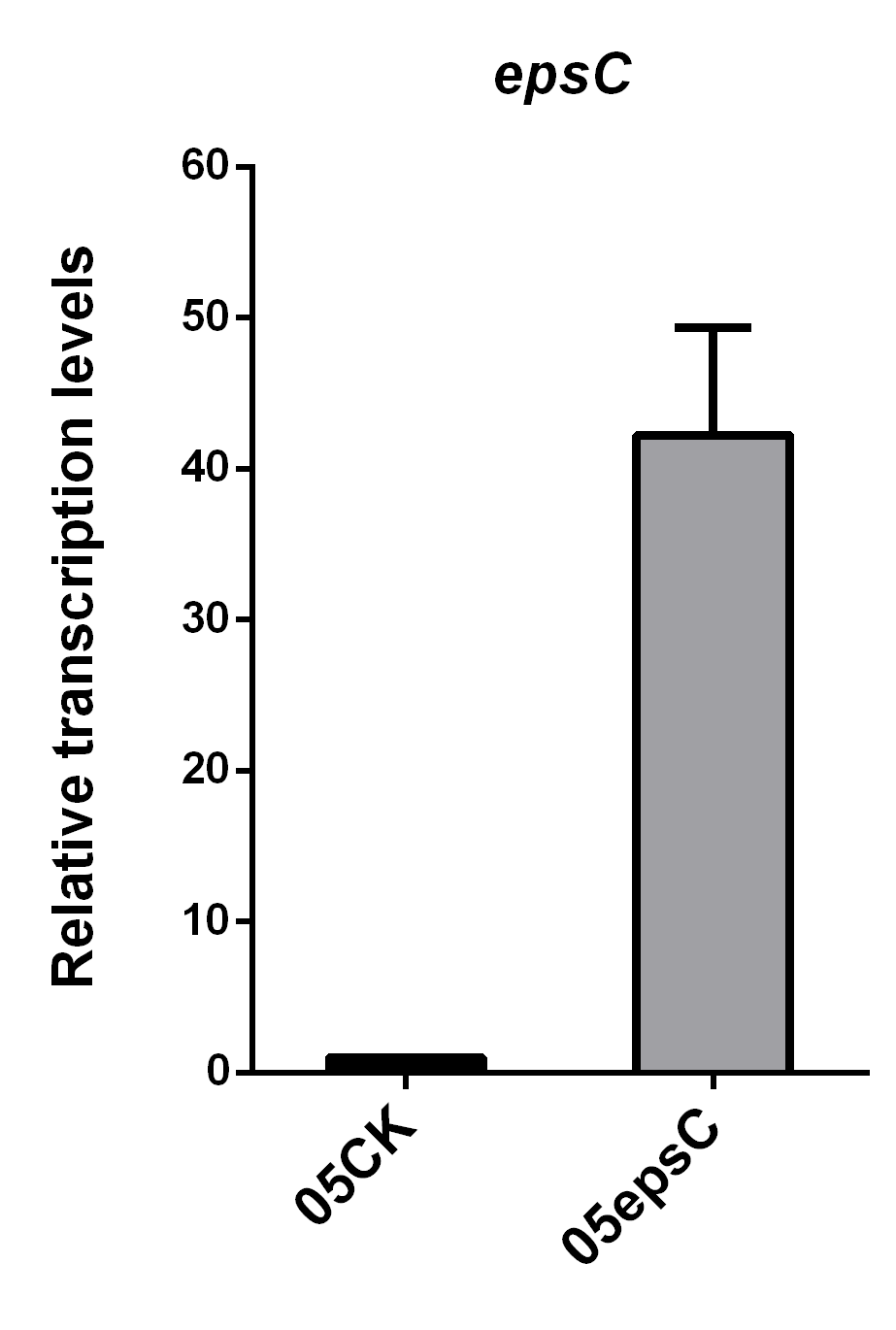


**Figure S4**. RT-qPCR analysis of the transcription level of *epsC* in *S. thermophilus* 05epsC in yoghurt. The fold change calculated was relative to the transcript levels in *S. thermophilus* 05epsC compared with that in 05CK. They were normalized using 16S rRNA gene as an internal control. Data are reported as mean ± SD from three independent experiments.

# Reference

Larkin, M.A., Blackshields, G., Brown, N.P., Chenna, R., McGettigan, P.A., McWilliam, H., et al. (2007). Clustal W and Clustal X version 2.0. *bioinformatics* 23(21)**,** 2947-2948.
